# Pharmacokinetic Imaging with Radiolabeled Molecularly Targeted Anticancer Drugs

**DOI:** 10.2967/jnumed.119.236174

**Published:** 2020-02

**Authors:** Martin Bauer, Markus Zeitlinger, Oliver Langer

**Affiliations:** * Medical University of Vienna Währinger-Gürtel 18-20 Vienna, Austria 1090 E-mail: martin.m.bauer@meduniwien.ac.at

**REPLY:** We would like to thank van de Stadt and colleagues for their positive and encouraging comments regarding our study recently published in *The Journal of Nuclear Medicine* (1). The central nervous system (CNS) is a pharmacologic sanctuary site in tumor treatment, due to the expression of ABCB1 and ABCG2 at the blood–brain barrier (BBB), which effectively restricts the brain distribution of most currently known anticancer agents. In our work, we have provided one possible mechanistic explanation for the improved efficacy of supratherapeutic dose erlotinib in the treatment of non–small cell lung cancer (NSCLC) CNS metastases, which is partial saturation of ABCG2- and ABCB1-mediated efflux transport of erlotinib at the BBB (1). This nonlinearity in erlotinib brain distribution may be exploited to achieve higher brain exposure of erlotinib itself or of concomitantly administered anticancer drugs, which are subject to efflux transport by ABCG2 and ABCB1 at the BBB. However, this increased CNS exposure comes at the expense of an increased incidence of adverse events (skin toxicity). Moreover, there may be potentially an increased risk of transporter-mediated interactions with concomitantly dosed drugs in other tissues, such as in excretory organs.

In fact, erlotinib is not the only molecularly targeted anticancer agent that is a substrate of ABCB1 and ABCG2. Numerous studies have shown that virtually all known kinase inhibitors are transported by ABCB1 and ABCG2 at the BBB. One option may be the combined use of these agents with ABCB1/ABCG2 inhibitors, which, however, due to reasons outlined above, will most likely not be a feasible option in clinical practice. Therefore the goal should be the development of potent new molecularly targeted anticancer drugs with good passive permeability and no affinity to ABCB1/ABCG2 to achieve high CNS exposure. This need has been recognized by pharmaceutical companies, leading to the development of new-generation kinase inhibitors with improved CNS exposure (2). One such example is osimertinib, which—despite being a substrate of ABCB1 and ABCG2—was shown to achieve substantially higher CNS exposure than the first-generation epidermal growth factor receptor (EGFR)–targeted tyrosine kinase inhibitor gefitinib (3). Indeed, osimertinib has proven more effective than gefitinib or erlotinib in treating patients with NSCLC brain metastases. Another example is AZD3759, which seems to be entirely devoid of ABCB1/ABCG2 affinity and which showed encouraging results in phase 1 clinical trials in patients with brain metastases (2). In the development of such drugs, PET imaging potentially plays an important role. Most of these agents can be radiolabeled without changing their chemical structures, and PET can be used to measure total brain concentrations in humans. In combination with in vitro assays to determine the extent of protein binding in the brain (e.g., brain slices or homogenates), the unbound concentrations of these agents in the human brain, which are most relevant for the pharmacodynamic effect, can be derived from the PET data (2).

Another potential application would be the use of PET to assess heterogeneities in the permeability between the BBB and the blood-brain tumor barrier (BBTB). There is evidence that brain tumors or metastases display a focally disrupted BBTB, which could lead to enhanced brain tumor delivery of anticancer agents as compared with healthy brain tissue. In clinical practice, BBTB disruption is assessed based on MRI measurements with gadolinium-containing contrast agents. However, these contrast agents possess physicochemical properties distinct from molecularly targeted anticancer agents and are not likely to be transported by ABCB1 and ABCG2. Therefore, brain delivery of MRI contrast agents may not be representative of brain delivery of molecularly targeted anticancer agents. Consequently, PET using the radiolabeled anticancer agents themselves in tumor patients is clearly a powerful approach, as illustrated by some recent studies (4,5).

In summary, the use of PET with molecularly targeted anticancer agents, which has been pioneered by the group at the VUmc in Amsterdam, has great potential to address and refine pharmacokinetic issues related to the design of brain-targeted anticancer drugs.
